# Complete Genome Sequence of *Escherichia coli* NCM3722

**DOI:** 10.1128/genomeA.00879-15

**Published:** 2015-08-06

**Authors:** Steven D. Brown, Suckjoon Jun

**Affiliations:** Department of Physics and Section of Molecular Biology, University of California–San Diego, La Jolla, California, USA

## Abstract

*Escherichia coli* NCM3722 is a prototrophic K-12 strain with robust physiologic phenotypes. We report the complete 4,678,045-bp chromosome and 67,545-bp F-like plasmid of this unique model organism.

## GENOME ANNOUNCEMENT

*Escherichia coli* is a landmark pathogen, and the K-12 strains are ubiquitous model organisms. *E. coli* NCM3722 is a prototrophic K-12 strain first described by Sydney Kustu and colleagues for studies of microbial physiology (1, 2). This strain exhibits unique phenotypes, including galactose metabolism and response to spectinomycin, that are more robust than those of the popular MG1655/W3110 lineage. To enable development of this strain as a model organism, we have sequenced the NCM3722 genome as well as a derivative, SJ358, which has been cured of the F plasmid.

*E. coli* NCM3722 was obtained from the Coli Genetic Stock Center (CGSC) (strain 12355). Cells were prepared for sequencing by streaking on nonselective media and culturing a single colony in LB supplemented with 0.2% glucose and 1× M salts (3). Genomic DNA was prepared by collection of cells by centrifugation from an exponential-phase culture and extraction with a Life Technologies PureLink genomic DNA minikit. A library for sequencing by Pacific Biosciences SMRT technology was prepared and sequenced with P6-C4 chemistry by the UCSD Institute for Genomic Medicine. Illumina 250-cycle paired-end libraries were similarly prepared from single-colony cultures collected in late exponential phase. The resulting single-molecule real-time (SMRT) and paired-end reads were jointly assembled with SPAdes v. 3.1.0 (4), which produced a contig of 4,678,045 bp corresponding to the chromosome and a contig of 67,545 bp corresponding to an F-like plasmid. Samplings of individual single-nucleotide variants (SNVs) were verified by PCR and Sanger sequencing. The F-like plasmid was modified by λRed recombineering to sequentially remove the *ccdB* gene and the predicted *rep-srnBʹ* genes. The resulting strain was serially subcultured in LB plus 10% SDS, and samples were periodically streaked on LB for single clones (5). Genomic DNA from one clone, SJ358, was prepared for short-read sequencing identically to NCM3722 and aligned to the NCM3722 contigs using breseq 0.26.0 (6) and Bowtie2 2.2.5. SJ358 is available from Addgene (http://www.addgene.org/67755/).

The chromosomal and F sequences corroborate SNVs and large differences revealed by prior 454-based sequencing, including phage lambda lysogeny and absence the Qin prophage and a CP4-6 prophage fragment. The *ilvG* and *rph-1* mutations of MG1655 are absent, and the *rpoS* gene is the 33Am variant from EMG2. We found a variety of new SNVs within the rRNA genes *rrsH*, *rrlG*, *rrlE*, *rrlB*, *rrlA*, and *rrlC*. A deletion that includes the *rfb* operon has orphaned the *rfbA* gene, resulting in an accumulation of SNVs. Deletion of the *rfb* operon has been associated with increased resistance to antibiotics, which may contribute to the phenotype of the strain (7).

### Nucleotide sequence accession numbers.

The complete genome sequence of the *E. coli* NCM3722 chromosome was deposited in GenBank under the accession number CP011495, and the F-like plasmid was deposited under the accession number CP011496. 
